# EAGERNESS OF NURSING HOME RESIDENTS TO INTERACT WITH ROBOT TEMI

**DOI:** 10.1093/geroni/igae098.2428

**Published:** 2024-12-31

**Authors:** Zeynep Abul, Sakeena Raza, Lynn McNicoll

**Affiliations:** Northeastern University, Boston, Massachusetts, United States; Brown Medicine, Providence, Rhode Island, United States; Brown University Medical School, Providence, Rhode Island, United States

## Abstract

Loneliness and social isolation existed among older adults before the COVID-19 pandemic, worsened due to lockdown, and visiting policies in nursing homes (NH) during pandemic. The aim of this study is to improve the interaction between robot Temi USA, Inc and NH residents. This is the preliminary finding of a quality improvement (QI) project. We conducted in-depth interviews with 54 long-term care NH residents to better understand their expectations from the self-navigating, autonomously driving humanoid robot temi. We asked the following focused question “If you wave a magic wand, what are some activities you would like to do with the robot?” and noted the answers. 5.6 % of the residents (n=3) responded neutrally by stating “I do not know “or “can’t think of anything”. Sixty-seven percent of the NH residents(n=36) responded with requests for interaction with temi. The most common request for interaction was “playing” chess, cards, piano, pool, baseball, Yahtzee, Bingo, and Wordhunt (n=20 out of 36; 56%).The remaining residents wanted to engage with temi by going outside (n=5), having temi sing with them (n=4); drawing with them (n=3); exercising with them (n=2); or just having a conversation with them (n=2). Fifteen residents (28%) wanted to have no interaction with temi. The high number of responses requesting interaction may imply that NH residents have an eagerness to interact with the robot. We will use those results to program an intervention to relieve loneliness of the NH residents for ongoing QI projects.

